# Two-Dimensional Post-Traumatic Measurements of Orbital Floor Blowout Fractures Underestimate Defect Sizes Compared to Three-Dimensional Approaches

**DOI:** 10.3390/tomography9020047

**Published:** 2023-03-05

**Authors:** Juergen Taxis, Lena Ungerboeck, Mika R. Gehrking, Constantin Motel, Matthias Wurm, Alexander W. Eckert, Gerrit Spanier, Felix Nieberle, Natascha Platz Batista da Silva, Nils Ludwig, Johannes K. Meier, Tobias Ettl, Torsten E. Reichert, Steffen Spoerl

**Affiliations:** 1 Department of Cranio- and Maxillofacial Surgery, University Hospital Regensburg, Franz-Josef-Strauß-Allee 11, 93053 Regensburg, Germany; 2 Department of Cranio- and Maxillofacial Surgery, Paracelsus Medical University Nuremberg, Breslauer Straße 201, 90471 Nuremberg, Germany; 3 Department of Radiology, University Hospital Regensburg, Franz-Josef-Strauß-Allee 11, 93053 Regensburg, Germany

**Keywords:** orbital floor fracture, blowout fracture, 2D fracture measurement, 3D fracture measurement

## Abstract

Orbital floor fractures represent a common fracture type of the midface and are standardly diagnosed clinically as well as radiologically using linear measurement methods. The aim of this study was to evaluate the accuracy of diagnostic measurements of isolated orbital floor fractures based on two-dimensional (2D) and three-dimensional (3D) measurement techniques. A cohort of 177 patients was retrospectively and multi-centrically evaluated after surgical treatment of an orbital floor fracture between 2010 and 2020. In addition to 2D and 3D measurements of the fracture area, further fracture-related parameters were investigated. Calculated fracture areas using the 2D measurement technique revealed an average area of 287.59 mm^2^, whereas the 3D measurement showed fracture areas with a significantly larger average value of 374.16 mm^2^ (*p* < 0.001). On average, the 3D measurements were 1.53-fold larger compared to the 2D measurements. This was observed in 145 patients, whereas only 32 patients showed smaller values in the 3D-based approach. However, the process duration of the 3D measurement took approximately twice as long as the 2D-based procedure. Nonetheless, 3D-based measurement of orbital floor defects provides a more accurate estimation of the fracture area than the 2D-based procedure and can be helpful in determining the indication and planning the surgical procedure.

## 1. Introduction

In the field of cranio-maxillofacial traumatology, fractures of orbital structures represent a common fracture type of the midface and account for up to 40% of craniofacial traumas [1,2,3,4]. Fractures of the orbital floor are accompanied by increased risks of diplopia and entrapment of the eye muscles, as well as enophthalmos as a long-term consequence [4,5,6,7]. In combination with a possible increase in orbital volume, the fracture that has occurred may alter the position of the eye, limit bulbar motility, and cause hypesthesia in the supply area of the infraorbital nerve [8,9]. Fractures of the orbital floor can be divided into the following main types of fractures, namely an isolated type, a zygomatico-orbito-maxillary fracture, and a complex panfacial type of fracture [1]. Regarding the isolated type, Smith et al. coined the term blow-out fracture [10]. In this case, a traumatic event transfers the force to the orbital floor as the first structure, which fractures while preserving the surrounding bony areas. This may result in herniation of soft tissue into the maxillary sinus. Especially in pediatric patients, a trap-door phenomenon may occur, leading to incarceration of the orbital soft tissue and the inferior rectus muscle due to the elasticity of the juvenile bone [11,12,13,14]. To avoid or even eliminate such conditions, surgical treatment of the orbital floor fracture aiming to restore the anatomical situation with an orbital implant is considered a standard procedure [7,15]. Before surgery, clinical evaluation of the symptoms as well as radiological estimation of the orbital floor defect are obligatory [16,17]. In this context, computed tomography (CT) has become more established and has replaced ordinary radiography [18,19,20]. Hereby, the defect sizes of orbital floor fractures can be assessed using two-dimensional (2D) as well as three-dimensional (3D) approaches. In previous studies, measurements were performed linearly and two-dimensionally based on computed tomography scans, and specific formulas were used for simplification and averaging the defect sizes [6,21]. Recent studies have been devoted to assessing defect sizes using 3D methods prior to fabrication of individual configurated implants [9,22,23]. In this regard, one type of reconstruction for orbital floor defects is noteworthy: Computer-aided design (CAD) and computer-aided manufacturing (CAM) techniques can be applied to build so-called patient-specific implants (PSIs), which have revolutionized the treatment even of complex orbital floor defects [24]. It is worth noting that this type of reconstruction for orbital floor defects demands not only more time prior surgery but also requires a certain level of technical experience. Design of a PSI based on CAD/CAM techniques either uses 3D data of the contralateral orbita, which is consecutively mirrored, or relays to special databases that provide 3D data for different orbital sizes and variations [24]. As a disadvantage of this technique, the timespan from PSI design until availability of the implant can be expected to be around five working days [25]. Usually, a PSI is built by selective laser welding methods. However, implants for reconstruction of the fractured orbital floor have increasingly been fabricated using a 3D printer or accomplished intraoperatively by bending the mesh grid onto previously printed models of the affected bony orbital structure [26,27,28]. Nonetheless, today, most orbital floor fractures are still diagnosed by eye based on CT, and surgical treatment is performed conventionally using implants, such as titanium meshes or polydioxanone foils (PDS), for reconstruction. In this regard, the ideal type of orbital floor reconstruction is still under debate, and material selection for orbital floor reconstruction varies remarkably between different institutions since there are no generally accepted guidelines [29]. Furthermore, besides selection of the ideal type of orbital floor reconstruction, identification as well as quantification of distinct fracture areas are also crucial in pre-surgical examination and for indicating surgical interventions. Hence, it is surprising that there has been little preliminary work on how defect measurement using the linear 2D method differs from the 3D-based approach. Today, there is an urgent need to compare 2D- and 3D-based methods to find the most accurate technique for defect size measurements. To the best of our knowledge, there has only been one prior related study, which compared 2D- and 3D-based approaches for defect size measurements in a small cohort of human cadavers and artificially created fractures [8]. In addition, another study described the differences in diagnostic accuracy between 2D and 3D images, proposing that the 2D method is significantly faster, and in some cases, more precise [30]. The most accurate estimation of the extent of the defect can provide the best guidance regarding the necessity of surgical treatment or the use of a conservative approach. Furthermore, a suitable fitting material and size can then be selected preoperatively in the event of a surgical intervention [31].

Therefore, the purpose of this work was to evaluate the accuracy in diagnostic measurement of isolated orbital floor fractures when using 2D and 3D measurement techniques.

## 2. Materials and Methods

### 2.1. Patient Selection and Data Collection

This retrospective study was performed at the Department of Cranio- and Maxillofacial Surgery, University Hospital Regensburg, Germany, and at the Department of Cranio- and Maxillofacial Surgery, University Hospital of the Paracelsus Medical University Nuremberg, Germany. A total of 576 patients, who underwent surgical treatment for a diagnosis of orbital floor fracture over a 10-year period (2010–2020), were evaluated. Clinical data such as age, sex, cause of fracture, timing of surgical intervention, duration of surgery, type of supply, and length of hospital stay were included in the analysis. Surgical supplies were applied using a PDS foil, a titanium mesh, or a PSI. When a PDS foil was used, the foil thickness was an additional parameter that was taken into account. The most important criteria for subsequent consideration of patient data were the presence of an isolated orbital floor fracture and at least two high-resolution CT scans, one pre-operative and one post-operative. The CT scans needed for DICOM data extraction and secondary measurements were coronal and sagittal reformations of the midface (bone window) with a slice thickness of 0.75 mm, and an additional axial scan for the 3D-based measurement. This reduced the final number of patients who were eligible for further segmentation and measurement of the fracture defect to 177.

### 2.2. 2D-Based Measurement of Orbital Floor Fractures

**Figure 1:**
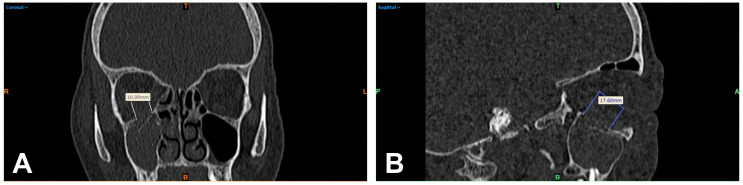
Multiplanar reformation of a patient’s midface (bone window) after orbital trauma: linear 2D measurement of the visible orbital wall defect of the right orbita in mediolateral extension in the coronal reformation (**A**) and anteroposterior extension in the sagittal image (**B**). R = right; T = top; L = left; colored B = bottom.

The DICOM data of the basic CT scans collected from patients were acquired and processed using Mimics image processing software (Mimics Innovation Suite 21.0, Materialise, Leuven, Belgium). For 2D measurement, the defect seen on CT was assessed linearly by two independent examiners (J.T. and L.U.), the obtained results were averaged, and the time required for this was recorded. First, the slice thickness d of the CT was determined. Then, the number n of CT slice images in which the orbital floor defect was evident was determined. Finally, the fracture area was measured based on the mediolateral extension (with a ruler tool integrated in the software (Figure 1A,B)) for later processing, then calculated using the following formula in approximation with and according to the method previously described by Ellis et al. [6]:

$$A=n_{anteroposterior}\times d_{CT}\times \frac{\sum l_{mediolateral}}{n_{anteroposterior}}$$

A= fracture area

$n_{anteroposterior}=$ number of slice images in anteroposterior extension

$d_{CT}=$ layer thickness of CT

$l_{mediolateral}=$ fracture range in mediolateral extension

### 2.3. 3D-Based Measurements of Orbital Floor Fractures

**Figure 2:**
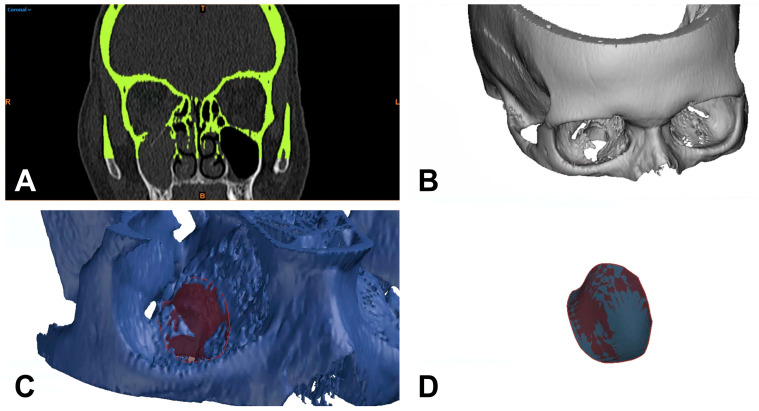
Separation of bony structures using thresholding of Hounsfield units and virtual tissue dissection (**A**), generation of a 3D model (**B**), and construction of a polynomial surface circularly around the defect for 3D-based defect measurement (**C**,**D**). R = right; T = top; L = left; colored B = bottom.

For the 3D-based measurement, the CT images were color-coded to differentiate areas in the trauma zone from non-involved parts of the scanned midface. The differentiation was based on thresholding of Hounsfield units (HU) using the software mentioned above. Subsequently, a virtual tissue dissection was performed and reduced to the bony structures (Figure 2A). A 3D model was generated from the bone selected by this procedure and exported as an STL (Standard Triangle Language) file for 3D analysis (Figure 2B). The STL files were then processed using GOM Inspect Pro (GOM Inspect Suite 2022, Carl Zeiss GOM Metrology GmbH, Braunschweig, Germany). For this purpose, the defect area was circularly marked on the upright bony orbital floor, and a polynomial area of the defect area was constructed. Subsequently, the resulting defect area within this polynomial could be calculated using the function integrated in the program (Figure 2C,D). The 3D measurement was again performed independently by two investigators (J.T. and L.U.) and the time required for this was noted.

### 2.4. Statistical Analysis

The statistical analysis was performed using IBM SPSS Statistics 26.0 (IBM Corp., Armonk, NY, USA) and GraphPad Prism 9.0 (GraphPad Software, La Jolla, CA, USA). The median (MED), mean (MV), and standard deviation (SD) of the 2D as well as 3D measurements were calculated. A paired Mann–Whitney U test was used to analyze the differences between the 2D- and 3D-based approaches. The significance level was defined as *p* < 0.05.

## 3. Results

### 3.1. Characterization of the Patient Cohort

During the observation period of 10 years, 177 patients fulfilled the previously mentioned criteria. A total of 60 patients (33.9%) were female, and 117 patients (66.1%) were male. The mean age was 49.1 years (range: 14–87 years) and the most common cause of fracture was a fall event with 66 cases (37.3%), followed by traffic accidents with 36 cases (20.3%), and rough offense with 35 cases (19.8%). On average, surgical intervention occurred after 5.68 days (ranging from 0 to 22 days) and the duration of surgery was 109.84 min (ranging from 23 to 550 min). The majority of patients were treated using a PDS foil (119 cases; 67.2%). Foil thicknesses of 0.15 and 0.25 mm were commonly used, with the 0.25 mm thickness being slightly more frequently utilized (51.4%). The inpatient stays of the surgically treated patients were on average 11.25 days (ranging from 3 to 61 days). However, surgical intervention was frequently delayed by several days due to post-traumatic swelling. The overall characteristics of the patient cohort are shown in Table 1.

### 3.2. Comparison of Orbital Floor Defect Areas Based on 2D and 3D Measurements

**Figure 3:**
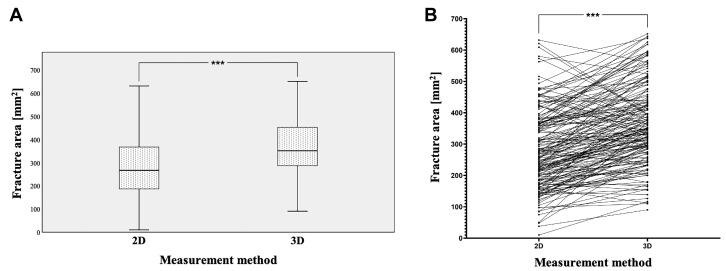
Using 2D and 3D measurement techniques, the calculated fracture areas (**A**) and connecting lines between individual measurements (**B**), with a statistically highly significant difference (*** *p* < 0.001).

The fracture areas calculated based on the 2D measurement technique had an average area of 287.59 mm^2^ with a standard deviation of 138.60 mm^2^ (minimum 10.00 mm^2^ and maximum 631.12 mm^2^). In contrast, using 3D-based measurements revealed fracture areas with a much higher average value of 374.16 mm^2^ and a standard deviation of 139.37 mm^2^ (minimum 90.46 mm^2^ and maximum 665.06 mm^2^). Here, the paired Mann–Whitney U test revealed a statistically highly significant difference between the 2D- and 3D-measured fracture areas (*** *p* < 0.001, Figure 3A,B). In the paired analysis, a total of 145 fractures were measured as larger when using the 3D-based approach, whereas only 32 fractures appeared to be smaller when using the 3D-based approach compared to 2D-based measurements (Figure 3B). To further explore this aspect, the difference was calculated for each individual patient, revealing a 1.53-fold difference between the 2D- and 3D-based measurements (SD: 1.06-fold: minimum 0.63-fold and maximum 11.7-fold). In summary, these data demonstrate that the area of fractures is significantly underestimated when using a 2D-based measurement approach. All results are listed in Table 2.

### 3.3. Comparison of Processing Duration of 2D- and 3D-Based Assessments of Orbital Floor Defect Areas

Given that the processing of 3D-based measurement is a more complex procedure than that of the 2D-based measurement, we compared the duration of both procedures as an additional parameter. The 2D measurement of the fracture surface took an average of 11 min (minimum of 5.4 min and maximum of 13.2 min), whereas the 3D measurement, including the previous processing of the CT datasets, took an average of 20 min (minimum of 18.7 min and maximum of 26.5 min). Therefore, we can conclude that the 3D-based assessment of defect areas takes approximately twice as long as the 2D-based analysis. All results are listed in Table 3.

## 4. Discussion

The topographical location and quantification of the surface area size of orbital floor blowout fractures is a primary factor in initial treatment decision-making and in the selection of appropriate orbital implants if surgical repair is indicated [32]. The development of clinically applicable and accurate workflows for determining orbital floor defect areas is therefore ongoing in order to improve surgical outcomes. In this study, we compared two approaches to measuring the defect area, one based on 2D assessments and one based on 3D assessments. The fracture areas of the isolated orbital floor fractures in our patient cohort had a mean value of 287.59 mm^2^ when using a 2D-based measurement approach, which represents values partially consistent with previous publications [8,33,34]. In contrast, the 3D-based measurements revealed significantly higher values (374.16 mm^2^ on average). Most likely, the 3D-based approach more accurately and realistically captures the area of the fracture than 2D-based measurements due to its more precise assessment of the structurally complex fracture configuration. In this case, the 3D-based measurements were approximately 1.53-fold larger compared to the 2D-based measurements; thus, it could be speculated that after a classical 2D measurement of an orbital floor fracture, one could add the average difference between 2D and 3D measurement (86.57 mm^2^) and thus obtain an approximately similar fracture area to a more accurate 3D measurement. However, not every 3D measurement turned out to be larger, which can be attributed to different fracture configurations, though fractures were, in general, more precisely evident in three dimensions than in the individual sectional images. In this regard, a noteworthy limitation of the study was its retrospective character; as basic, thin-sliced DICOM data of necessary CT examinations with ≤1 mm slice thickness were only accessible for 3 days after the examination was performed in our hospital, as long-term archiving of the CT examinations in the local PACS was mostly performed for diagnostic axial scans with a slice thickness ≥1 mm. That limitation affected the accuracy of fracture measurement. 

The more accurate 3D measurement option presented here can sometimes provide guidance when making decisions in difficult or borderline cases regarding the need for surgical intervention. The decision of whether to proceed with a conservative or surgical procedure is classically made based on clinical impairments, such as diplopia or a sensitive deficiency of the infraorbital nerve. In this regard, radiographic criteria that determine the need for surgical interventions are still controversial [16,33,35]. Certainly, complications such as orbital muscle entrapment as well as a retrobulbar hematoma represent a non-questionable indication for immediate surgical intervention. However, in most orbital floor defects, these kinds of complications are not applicable, which raises questions about possible clinical as well as radiological decision-making aids to indicate a surgical intervention. The extent of a potential orbital floor defect represents a major factor in this regard: Based on the extent of distinct orbital floor defect areas, application of a PDS foil, a conventional titanium mesh, or a PSI is indicated. Whereas smaller defects up to a defect area of 2.5 cm^2^ are predominantly reconstructed with a PDS foil, CAD/CAM techniques provide the basis for reconstruction of larger orbital floor or combined orbital floor and wall defects using a PSI [24]. A potentially valuable additional opportunity could arise from the development of a fully automated algorithm and use of artificial intelligence, which would allow 3D diagnostics of the fracture surface to be implemented in less time, thereby greatly facilitating and accelerating patient-specific fabrication of the restorative material [23]. Our retrospectively collected and well-characterized patient cohort represents an opportunity for the development of such automated procedures using the cohort as a learning platform for the artificial intelligence. However, more extensive and independent cohorts will also be necessary to validate the concept of using artificial intelligence for the assessment of orbital floor fractures.

The greatest limitation of this study was the subjectivity of the examination since both the 2D measurement and the 3D measurement methods were limited by the individual measurement accuracy of the examiners. Furthermore, the extent of the fracture could only be recorded as accurately as it could be represented and recorded in the CT images. In addition, the 2D measurements, which were not perfectly accurate due to the approximate nature of the calculation formula, were intended to simulate the classic clinically used procedure for diagnosing an orbital floor fracture using broadly available cross-sectional imaging techniques. In everyday clinical practice, the diagnostic preliminary work is usually conducted by a resident, while the indication for surgical treatment is usually given by a specialist. The preliminary work of Faisal et al. could represent a possible and more objective approach compared to manual area measurement [36].

Furthermore, our work showed that 2D measurement of the fracture took on average 11 min and thus could be performed clearly faster than the 3D measurement, which, after some training and when including prior segmentation of the CT dataset, took at least 20 min, twice the time. This result is similar to the time outcomes of Ploder et al. and Jarrahy et al. [8,30]. This is partly a reason why 3D fracture measurement in an acute diagnostic case appears to be impractical as it is time-consuming, meaning 2D assessment of the fracture is still performed [37]. 

Finally, the retrospective nature of this study must be mentioned as a limitation. Due to ethical considerations, only limited clinical data available could be processed. Additionally, the temporal difference between the fracture event and the imaging obtained in the treatment course had a certain influence on the assessability of the fracture size because after a certain time, bony structures lying in the fracture area can no longer be identified as such. This favors an assessment of the fracture area as smaller than is correct, although this affects both 2D and 3D assessment equally.

In conclusion, it seems a consideration of the volume change of pre- and post-operative orbital floor fractures will be useful in future studies. Since the orbital volume is inevitably subject to change after reconstruction [9,38], new insights may be gained in this regard by using 3D measurement methods and including clinical outcomes.

## 5. Conclusions

CT-based 3D measurements of orbital floor defects revealed statistically significant larger values compared to the application of the clinically used 2D-based assessment. The study results show that 3D-based measurements capture defect areas more realistically, which ultimately impacts decision-making and planning of surgical interventions. In addition, 3D measurements appear to be the more suitable aid when deciding between a surgical or conservative procedure.

## Figures and Tables

**Table 1:** Clinicopathological characteristics of the patient cohort.

Category	MV (Range)	Total (*n* = 177)
Gender:		
Female	-	60 (33.9%)
Male	-	117 (66.1%)
Age (years)	49.10 (14–87)	-
Cause of fracture:		
Rough offense	-	35 (19.8%)
Fall	-	66 (37.3%)
Sports accident	-	14 (7.9%)
Traffic accident	-	36 (20.3%)
Horse kick	-	5 (2.8)
Other	-	21 (11.9%)
Surgery after fracture (days)	5.68 (0–22)	-
Surgery duration (minutes)	109.84 (23–550)	-
Supply type:		
PDS foil	-	119 (67.2%)
Titanium mesh	-	32 (18.1%)
PSI	-	5 (2.8%)
Maxillary sinus balloon	-	1 (0.6%)
Reposition only	-	17 (9.6%)
Refusal of supply	-	2 (1.1%)
Monocortical iliac crest	-	1 (0.6%)
PDS foil thickness: (mm)		
0.15	-	86 (48.6%)
0.25	-	91 (51.4%)
Inpatient stay (days)	11.25 (3–61)	-

PSI = patient-specific implant; PDS = polydioxanone; MV = mean value.

**Table 2:** Fracture areas analyzed based on 2D- and 3D-based measurement methods and the difference between the two.

*n* = 177	Measurement Method (mm^2^)		Difference (-Fold)
	2D	3D	
Mean	287.59	374.16	1.53
Median	267.12	351.90	1.35
SD	138.60	139.37	1.06
Minimum	10.00	90.46	0.63
Maximum	631.12	665.06	11.7
*p*-value	<0.001		-

2D = two-dimensional; 3D = three-dimensional; SD = standard deviation.

**Table 3:** Processing times of the 2D- and 3D-based measurement methods of orbital floor defect areas.

*n* = 177	Processing Time (Minutes)	
	2D	3D
Mean	11	20
Minimum	5.4	18.7
Maximum	13.2	26.5

2D = two-dimensional; 3D = three-dimensional.

## Data Availability

Data can be obtained from the clinicians that conducted the work independently on request. Data are not stored on publicly available servers.
